# Middle Range Theory of Traumatic Childbirth: The Ever-Widening Ripple Effect

**DOI:** 10.1177/2333393615575313

**Published:** 2015-03-18

**Authors:** Cheryl Tatano Beck

**Affiliations:** 1 University of Connecticut, Storrs, Connecticut, USA

**Keywords:** childbirth, mothers, mothering, postpartum care, posttraumatic stress disorder (PTSD), theory development, trauma

## Abstract

A middle range theory of traumatic childbirth was developed using Morse’s method of theoretical coalescence. The scope of this qualitative theory was increased by formalizing the connections between 14 individual studies all conducted by the same researcher on the same topic, with different groups, using different research designs and different types of analyses. Axioms were derived from this research program along with attributes of traumatic childbirth, posttraumatic stress, and secondary traumatic stress. This middle range theory addresses the long-term chronic consequences of a traumatic birth for mothers including its impact on breastfeeding, subsequent childbirth, and the anniversary of birth trauma. The impact on fathers and clinicians present at the traumatic birth is highlighted as secondary traumatic stress comes into play. Troubling glimpses of difficulties in mother–infant bonding are revealed.

“Just as ripples spread out when a single pebble is dropped into water, the actions of individuals can have far-reaching effects” (dalailamaquotes.net). Health care providers’ acts always have consequences, either positive or negative. These consequences can spread out like ripples when a stone is dropped into a pond. In a U.S. national survey, 9% of new mothers screened positive for meeting all the necessary *Diagnostic and Statistical Manual of Mental Disorders* (4th ed., text rev.; *DSM-IV-TR*; American Psychiatric Association [APA], 2000) criteria to be diagnosed with posttraumatic stress disorder (PTSD) secondary to traumatic childbirth. A meta-analysis of risk factors for postpartum PTSD revealed that the perceived quality of interactions with obstetrical care providers during labor and delivery was a significant predictor (Grekin & O’Hara, 2014). As one woman shared, “I am amazed that 3½ hours in the labor and delivery room could cause such utter destruction in my life. It truly was like being the victim of a violent crime or rape” (Beck, 2004a, p. 32). We need to ask what could have occurred to turn this woman’s birthing into a rape scene? What are the ripple effects of those hours in labor and delivery on a woman’s life?

Kounin (1970) first coined the term “ripple effect” to describe the positive effect a teacher may have on students. A ripple effect is “a spreading effect or series of consequences caused by a single action or event” (dictionary.com). We need to understand ripples because they are the first phase in the growth of bigger ocean waves (Zinker, 2013). We need to understand the ripple effect of traumatic childbirth to help prevent the growth of possible larger adverse consequences. To guide evidence-based practice, the method of theoretical coalescence was used to develop a middle range theory of traumatic childbirth and its ever-expanding ripple effects.

## Theoretical Coalescence

Theoretical coalescence is a method developed by Morse (in press), whereby a series of studies on a topic is combined into a whole to create a higher, more abstract-level middle range theory. The scope of qualitative theory can be increased by formalizing the connections between individual studies on the same topic that may have been conducted with different groups, different types of analyses, different types of qualitative inquiry in different areas, and different points in time. Because the researcher who has conducted this series of studies is privy to the intimate details of these qualitative data and has total access to these data, the possibility of cognitive sliding is decreased (Morse, in press). The likelihood of guessing about interpretations and meanings of data as the researcher tries to fit the findings together is lessened. The researcher has “3 D Vision of the Topic” and can ask and answer significant analytical questions of these data from the series of studies.

Theoretical coalescence consisted of six steps (Morse, in press):

1. Identifying significant concepts;
2. Evaluating the development of the concepts common to each study;
3. Diagramming the concepts and their position overall. In this step, the researcher maps each concept in position depending on the primary contribution to this higher-level middle range theory;
4. Identifying the attributes that are common to each example of the concepts. Look for common attributes between the concepts and link them laterally or horizontally;
5. Developing analytic questions about the nature of the overarching concept and the answers from each individual study and their attributes;
6. Diagramming and writing the higher-level middle range theory which overcomes the frequent criticism of small, individual qualitative studies.

As the researcher achieves each of the above steps in theoretical coalescence, each study is decontextualized to its basic processes and characteristics/attributes.

## The Development of the Theory of Traumatic Childbirth

The traumatic childbirth theory was developed from a synthesis of a decade of predominantly qualitative studies that I have conducted (Table 1). Synthesized were the data from eight descriptive phenomenological studies, two mixed-methods studies, one narrative analysis, one quantitative survey, one case study, and one secondary qualitative analysis. Types of qualitative data analyses included Colaizzi’s (1978) phenomenological analysis, Krippendorff’s (2013) content analysis of the qualitative strand in the mixed-methods studies, Burke’s (1969) narrative analysis, and Yin’s (2013) case study analysis.

**Table 1.** List of Traumatic Childbirth Studies.

Study 1. Birth trauma: In the eye of the beholder (Beck, 2004a)
Study 2. PTSD due to childbirth: The aftermath (Beck, 2004b)
Study 3. Pentadic cartography: Mapping birth trauma narrative (Beck, 2006a)
Study 4. The anniversary of birth trauma: Failure to rescue (Beck, 2006b)
Study 5. Impact of birth trauma on breastfeeding: A tale of two pathways (Beck & Watson, 2008)
Study 6. An adult survivor of child sexual abuse and her breastfeeding experience: A case study (Beck, 2009a)
Study 7. The arm: There is no escaping the reality for mothers of children with obstetric brachial plexus injuries (Beck, 2009b)
Study 8. Subsequent childbirth after a previous traumatic birth (Beck & Watson, 2010)
Study 9. PTSD in new mothers: Results from a two-stage U.S. national survey (Beck, Gable, Sakala, & Declercq, 2011)
Study 10. A mixed-methods study of secondary traumatic stress in labor and delivery nurses (Beck & Gable, 2012)
Study 11. The obstetric nightmare of shoulder dystocia: A tale from two perspectives (Beck, 2013)
Study 12. Fathers’ experiences of being present at their partners’ traumatic childbirth (Beck, Driscoll, & Watson, 2013)
Study 13. Mothers’ experiences of EMDR treatment for the posttraumatic stress symptoms (Beck, Driscoll, & Watson, 2013)
Study 14. Shaken belief in the birth process: A mixed-methods study of secondary traumatic stress in CNMs (Beck, LoGiudice, & Gable, 2015)

*Note.* PTSD = posttraumatic stress disorder; EMDR = eye movement desensitization reprocessing; CNMs = certified nurse- midwives.

All eight of the descriptive phenomenological studies and the one narrative analysis were conducted via the Internet. Women were recruited to participate in these studies through Trauma and Birth Stress’ (TABS) website (www.tabs.org.nz). TABS is a charitable trust located in New Zealand dedicated to support women who have experienced traumatic childbirths.

The study that began this systematic series of research focused on just what it is about childbirth that can be so traumatic that it can result in some women developing PTSD. The essential elements of traumatic childbirth were discovered and what was found was that, just like beauty, traumatic childbirth is in the eye of the beholder (Beck, 2004a). What obstetrical clinicians would consider a routine birth may be perceived as traumatic by the mothers. The next study looked specifically at PTSD due to birth trauma (Beck, 2004b). This study was a descriptive phenomenological study. A narrative analysis of traumatic childbirth followed because a different perspective was needed. Descriptive phenomenology divided up women’s stories for general themes to emerge. Now what was needed was to keep each story in its entirety for analysis, and that called for the use of narrative analysis (Beck, 2006a).

What is the chronic nature of traumatic birth? This was the focus of the next few studies in my program of research: the yearly anniversary of birth trauma (Beck, 2006b), the impact of birth trauma on breastfeeding (Beck & Watson, 2008), and subsequent childbirth following a previous traumatic birth (Beck & Watson, 2010). One of the women in the breastfeeding study who was a survivor of child sexual abuse shared such a powerful, detailed story of her traumatic birth that, with her permission, a case study was written (Beck, 2009a). Another offshoot of these prior studies of birth trauma was a secondary qualitative data analysis study that concentrated on a number of women with similar traumatic births, namely, shoulder dystocia (Beck, 2009b). Another study examined mothers’ experiences with eye movement desensitization and reprocessing (EMDR) treatment for their posttraumatic stress symptoms (Beck, Driscoll, & Watson, 2013).

What is the prevalence of women in the United States who experience posttraumatic stress symptoms due to childbirth? These quantitative data would supplement the prior qualitative studies. So with the help of Childbirth Connections, a U.S. national survey was conducted (Beck, Gable, Sakala, & Declercq, 2011). A sobering 9% of the sample of U.S. new mothers screened positive for meeting all the necessary *DSM-IV-TR* (APA, 2000) criteria to be diagnosed with PTSD.

**Figure 1.**
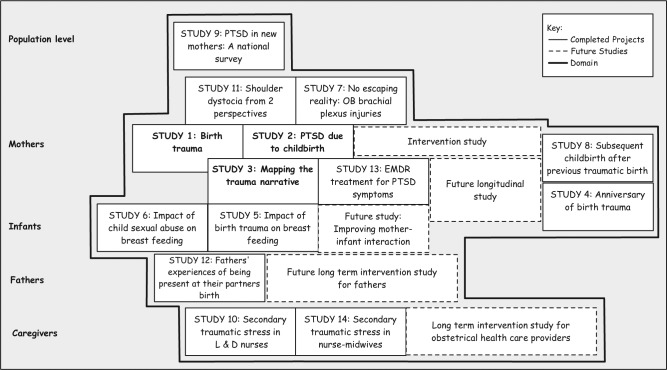
Research program conducted in the domain of traumatic birth syndrome. *Note.* PTSD = posttraumatic stress disorder; OB = obstetric; EMDR = eye movement desensitization reprocessing; L&D = labor and delivery.

My research program next extended to a different group other than mothers who had experienced birth trauma: fathers and obstetrical clinicians. One phenomenological study and two mixed-methods studies answered the question: Did traumatic births have an impact on fathers (Beck et al., 2013), on labor and delivery nurses (Beck & Gable, 2012), and on certified nurse-midwives (CNMs; Beck, LoGiudice, & Gable, 2015)? Located in Figure 1 is a diagram of the different types of groups included in my research program from the population level down to obstetric caregivers (Morse, 2001). This figure also identifies gaps where new data are needed. A series of interventions studies are needed for each group. Examples of these needed intervention studies can include (a) preventing traumatic childbirth in the first place, (b) decreasing posttraumatic stress symptoms in mothers once they do experience birth trauma, (c) decreasing posttraumatic stress symptoms in partners who are present during a traumatic birth, (d) decreasing secondary traumatic stress symptoms in obstetric caregivers who attend traumatic births, and (e) improving mother–infant interaction in dyads where the mother is experiencing posttraumatic stress symptoms.

## Axioms for the Theory of Traumatic Childbirth

Axioms were derived from my research program. Axioms are assumptions that are “held to be researchable, all things considered, and provided a context for the macro and micro analytic processes inherent in the theory” (Morse, in press)

1. Traumatic birth can be considered as an extreme traumatic stressor that can lead to PTSD.
2. Posttraumatic stress symptoms are a result of traumatic childbirth and can vary in intensity and duration.
3. Traumatic childbirth can have long-term, chronic consequences.
4. Traumatic childbirth can have stinging tentacles that can lash out at obstetrical clinicians and significant others who were present at the birth.
5. Posttraumatic stress symptoms can interfere with mother–infant interaction.
6. Traumatic birth can negatively affect a mother’s breastfeeding experience.
7. At the anniversary of a traumatic birth, posttraumatic stress symptoms can flair up.
8. Subsequent childbirth after a previous birth trauma is an anxiety-filled time for mothers.
9. Not all subsequent childbirths after a previous birth trauma are healing.

## Asking Analytical Questions of the Studies Themselves

1. What is it about childbirth that can be so traumatic?
2. What are the essential characteristics of posttraumatic stress due to birth trauma?
3. Are there long-term consequences of birth trauma for the mother?
4. Does a traumatic childbirth affect more than just the mother?
5. What strategies do women use to get through a subsequent pregnancy following a traumatic birth?
6. What is secondary posttraumatic stress due to attending a traumatic birth?

## What Is Birth Trauma?

Birth trauma is an event or events that occur at some phase during childbearing that involves the woman being stripped of her dignity and/or an actual or threatened serious injury to the mother or her unborn child. The mother may experience terror, helplessness, loss of control, powerlessness, or horror. Trauma can be psychological or physical. When women speak of their traumatic births they describe them as “feeling abandoned and alone,” “betrayed,” “raped,” “my dignity was taken from me,” “treated like a nothing,” and “terrified to the core of my being.”

## Attributes of a Traumatic Birth

As a concept, birth trauma consists of characteristics or attributes that are common to instances of what a woman perceives as a traumatic childbirth. These attributes are

1. Deprived of caring
2. Stripped of their dignity
3. Terrifying loss of control
4. Neglected communication
5. Buried and forgotten

### Deprived of Caring

During labor and delivery, is it too much to ask for women to feel cared for? In the case of a traumatic birth, the answer is yes. “Abandoned,” “alone,” “unsupported,” and “no reassurance” were words often used by mothers to describe how they felt during the birth process. When explaining the type of care they received during such a vulnerable time as labor and delivery, the following adjectives were shared: “cold,” “mechanical,” and “technical.” Women did not feel like they were acknowledged as individuals but instead felt like a “piece of meat on an assembly line.” “Please someone show me a little compassion; hold my hand.” As one woman shared, “Mommy is so sorry baby! No one is caring for us” (Beck et al., 2013, p. 16).

### Stripped of Their Dignity

Was it too much to ask for to be treated with respect during the birthing process? The answer for women who perceived their labor and delivery as traumatic was yes. According to the *Diagnostic and Statistical Manual of Mental Disorders* (5th ed.; *DSM-5*; APA, 2013), an extreme traumatic stressor that can lead to PTSD involves a situation in which the person perceived an actual or threatened death or serious injury to either oneself or to others. Stripped of a person’s dignity is not included as a possible extreme traumatic stressor. This, however, was a core characteristic involved in traumatic childbirth. For some women, they did not fear for their life or that of their unborn child, but instead were stripped of their dignity. They felt degraded and disrespected. Feeling raped on the delivery table instead of experiencing what should be one of the most precious events in a woman’s life was at the heart of birth trauma for some mothers.

### Terrifying Loss of Control

During labor and delivery was it too much to ask for women to have some semblance of control over what was happening to them? Again the answer was yes for women who experienced birth trauma. Women felt powerless and helpless. It was not a degree of but a total lack of control that ignited terror in the women. They felt like their bodies were seized and controlled by the labor and delivery staff. Women felt like their bodies, which were still carrying their precious unborn child, no longer belonged to them. While trying to cope with their increasingly strong contractions, women felt powerless to protest what was being done to their bodies. The only mode of survival was obedience. For some mothers they had no means of defense other than full surrender as they feared for their own safety and that of their unborn infant.

### Neglected Communication

Was it too much to ask for to be communicated with during the birthing process? Sadly, the answer was yet again, yes. Women felt invisible as procedures were being done to their bodies as if they were not present. One mother recalled that “the hospital staff discussed my baby’s possible death in front of me and argued in front of me just as if I weren’t there” (Beck, 2004a, p. 33). Lack of communication can create terror in such a vulnerable state. “Please, someone talk to me and tell me what is happening.” Women envisioned the worst-case scenarios without any information to guide their thinking.

### Buried and Forgotten

Was it too much to ask for to have anyone focus on what the woman had endured to give birth to her baby? Yes, it was too much to ask as everyone, family, friends, and clinicians, celebrated the safe arrival of a healthy newborn baby. Success was defined completely in terms of the outcome of the infant. Mothers’ traumatic experiences of labor and delivery were pushed into the background as a healthy baby took center stage. No one wanted to hear or dwell on all that the mother had endured to give birth. Repeatedly, mothers were told “the baby could have died, but is fine. Celebrate and move on.”

## Combinations of Attributes

Women did not need to experience all five of these core attributes to perceive these labor and delivery as traumatic. Any combinations of these characteristics can lead to birth trauma. Women were systematically stripped of protective layers leaving them vulnerable and fragile during labor and delivery. That is why one woman could have had a severe postpartum hemorrhage and be fine, whereas another woman suffering a postpartum hemorrhage could develop PTSD.

The attributes of traumatic birth just described emerged from phenomenological studies where general themes were the outcome. Use of a different qualitative method, narrative analysis, led to the discovery of other aspects of traumatic childbirth. Instead of fracturing mothers’ stories of birth trauma in phenomenology, now with narrative analysis, women’s stories were kept whole in their entirety. The sequencing of a series of events in the multiple scenes in traumatic childbirth revealed the repeated, multiple insults women endured during the birthing process (Beck, 2006a). As one woman vividly recalled “This is the birth of my child and I am feeling like a prisoner of war waiting for another rape” (Beck et al., 2013, p. 15). Birth trauma consisted of repeated psychological and/or physical abuse.

Traumatic childbirth was examined by three qualitative methods for confirmation of its essential attributes. The three methods included descriptive phenomenology (Beck, 2004a), narrative inquiry (Beck, 2006a), and secondary qualitative analysis (Beck, 2013; Table 2).

**Table 2.** Attributes of Traumatic Childbirth Confirmed by Three Qualitative Methods.

Characteristics	Phenomenology (Beck, 2004a)	Narrative Analysis (Beck, 2006a)	Secondary Analysis (Beck, 2013)
Deprived of caring	X	X	X
Stripped of dignity	X	X	X
Terrifying loss of control	X	X	X
Neglected communication	X	X	X
Buried and forgotten	X		

## What Is Posttraumatic Stress Following Birth Trauma?

Posttraumatic stress is the response to experiencing a traumatic event that triggers a cascade of symptoms reflecting significant distress and impairment in social interactions. Four clusters of behavioral symptoms can be involved: re-experiencing, avoidance, negative cognitions and mood, and arousal (APA, 2013). Intensity of these posttraumatic stress symptoms can range from mild to severe leading to PTSD in some mothers.

## Attributes/Characteristics

As a concept, posttraumatic stress consists of the following characteristics or attributes that are common to instances of what new mothers experience following birth trauma. These attributes are

1. Uncontrollable flashbacks/nightmares
2. Numbing detachment
3. Increased arousal: Seething anger, difficulty sleeping
4. Retreating from the world of motherhood

These attributes of posttraumatic stress response across studies are elaborated in Table 3. The types of qualitative methods used in these studies included descriptive phenomenology, case study, and secondary qualitative data analysis.

**Table 3.** Comparison of Posttraumatic Stress Attributes in Eight Studies.

Characteristics	PTSD (Beck, 2004b)	Anniversary (Beck, 2006b)	Breastfeeding (Beck & Watson, 2008)	Case Study (Beck, 2009a)	Subsequent Childbirth (Beck & Watson, 2010)	L&D Nurses (Beck & Gable, 2012)	Obstetric Nightmare (Beck, 2013)	CNMs (Beck, LoGiudice, & Gable, 2015)
Flashbacks/nightmares	X	X	X	X	X	X	X	X
Numbing detachment	X	X	X	X	X	X	X	X
Seething anger	X	X	X			X	X	X
Retreating from world of motherhood	X	X	X	X	X	X	X	X

*Note.* PTSD = posttraumatic stress disorder; L&D = labor and delivery; CNMs = certified nurse- midwives

### Uncontrollable Flashbacks/Nightmares

Presence of permanent “loop tracks” in their brains was how women described the uncontrollable re-experiencing of their traumatic births. Mothers explained how the distressing video of the birth was on automatic replay. Women did not have any control over when they would “go to the movies” and would be stuck in the past not being able to fully live in the present. After struggling with these flashbacks during the day, mothers did not get any solace at night. That was when the terrifying nightmares occurred, as one woman shared:

Like Lady Macbeth, I become terrified of sleeping! I would go without sleep for about 72 to 96 hours. I always knew I’d have to fight the nightmares again. I was scared that this time I wouldn’t have the strength to fight it, that it would succeed in destroying me. (Beck, 2004b, p. 219)

### Numbing Detachment

Devoid of all positive findings is how women explained how they went through their day mechanically taking care of their babies, just going through the motions. Women were only a shadow of their former selves. One mother admitted, “I’d wake up numb, unable to feel a thing. I’d drag myself through the day. I am having the hardest time trying to overcome this feeling of being dead” (Beck, 2004b, p. 220). Women detached themselves from others—baby, partner, other children, family, and friends. It did not matter who it was. Mothers could not connect closely with others. This can have a devastating effect on mother–infant interaction and bonding when the mother is not “emotionally available” for her infant at this crucial period.

### Increased Arousal: Seething Anger, Difficulty Sleeping

Taking the place of any positive, joyful emotions was seething anger. Mothers’ anger was directed in multiple directions: to the labor and delivery clinicians, to family members present at the birth, and also to themselves. Anger was aimed at the obstetrical staff that the mother had entrusted with the care and safety of herself and her baby. Women felt betrayed. Women also directed their anger toward their partners who were with them during labor and delivery. How could their partners just stand there during the traumatic birth and not step in to help them? Last, at times mothers turned their anger inward toward themselves. How could they have let this happen? Why did they trust the labor and delivery staff? This increased arousal also took the form of difficulty sleeping for the women. Compounding this sleeping problem were the terrifying nightmares that plagued mothers.

### Retreating From the World of Motherhood: Persevering Avoidance

Women persevered in trying to avoid the distressing reminders of their traumatic births, be that persons, places, thoughts, or activities. For example, women would go out of their way not to drive by the hospital where they gave birth even if it meant driving extra miles. Unwanted recollections of their birth trauma brought on the distressing flashbacks and nightmares. For these women, who was the daily reminder of their traumatic childbirth? Their infants. So for some mothers, they distanced themselves from their infants. The walls erected due to the birth trauma separated mothers and their infants. Women experiencing posttraumatic stress isolated themselves from other mothers and babies. Women’s dreams of the anticipated world of motherhood were scattered and eluded them.

## Are There Long-Term Chronic Consequences of a Traumatic Birth?

Four studies were designed in sequential order to explore this question using descriptive phenomenology and case study designs.

### Anniversary of Birth Trauma

Researchers have reported that anniversary time for survivors of a traumatic event, such as 9/11, involved a flair up of posttraumatic stress symptoms. Does this happen for women who experienced a traumatic childbirth? My assumption was that mothers would find the anniversary day difficult to get through. This assumption was correct but it did not go far enough into the impact of the anniversary for these mothers. Women revealed that it was not just the actual day of the anniversary that was distressing but there also was a build up during the weeks and months leading to the anniversary as well as a fragile, vulnerable time once the anniversary was over. The approaching anniversary plagued women with an array of distressing thoughts and emotions. Dread, anxiety, fear, sadness, and guilt were some of the frequent distressing emotions that came to the forefront as the anniversary drew closer. Clocks and seasons of the year played key roles in reminding mothers the anniversary was approaching.

What complicated matters for mothers was that the anniversary day was also their child’s birthday—a cause for celebration. This added another layer of difficulty for women who have experienced traumatic childbirth. For other types of trauma, such as a rape or motor vehicle accidents, the survivors do not have this conflicting anniversary where it is also a celebration of an event. Family and health care providers failed to rescue women yet again as they struggled to get through the anniversary. We failed to rescue them during their traumatic births and failed them again as no one acknowledged their struggles or offered support at anniversary time. Surviving the actual day of the anniversary took a heavy toll on mothers. They felt vulnerable and fragile as their wounds were reopened. Time to recuperate was needed.

### Breastfeeding

A descriptive phenomenological study (Beck & Watson, 2008) and a case study (Beck, 2009a) revealed the impact of birth trauma on mothers’ breastfeeding experiences which can lead them down two strikingly different paths: one facilitating and one impeding breastfeeding. Women shared that there were three factors that propelled them into continuing to breastfeed after their birth trauma: (a) proving themselves as mothers. They were determined to succeed at something related to motherhood; (b) making up to their infants for the traumatic way they were brought into the world; and (c) providing a temporary time out from the distressing thoughts of their traumatic births.

The other pathway that hindered women’s breastfeeding attempts was lined with the following negative impacts after birth trauma: (a) viewing their breasts as one more part of their body to be violated just as the women had felt violated during the birth, (b) enduring intense physical pain from the birth trauma, such as a shoulder dystocia delivery, (c) perception of insufficient milk supply, (d) intrusive flashbacks of traumatic birth while breastfeeding, and (e) disturbing detachment from their infants.

### Subsequent Childbirth After a Previous Traumatic Birth

The long-term detrimental effects of traumatic childbirth extended into yet another aspect of women’s lives: subsequent childbirth. To have more children or not. Some women decided that they would not get pregnant again. They refused to take the risk of another traumatic birth. A few women, who did want more children but could not face another labor and delivery, chose to use a surrogate mother. Some women did get pregnant again but it was unplanned, whereas other women did choose to risk another birth trauma because of their desire for more children. No matter whether the subsequent pregnancy was planned or not, the pregnancy was filled with fear, terror, anxiety, panic, denial, and dread as the women prepared to endure the 9 long months to face what they feared the most: another labor and delivery. The following quote captures this: “My 9 months of pregnancy were an anxiety filled abyss which was completely marred as an experience due to the terror that was continually in my mind from the experience 8 years earlier” (Beck & Watson, 2010, p. 245). What strategies do women use to survive the 9 months of pregnancy following a traumatic birth? Women shared various strategies that they had used to cope with pregnancy and the looming labor and delivery (Beck & Watson, 2010). Some of these strategies included

- Exercising to help baby get in the correct position
- Mapping out their pelvis
- Writing a detailed birth plan
- Painting birth art
- Avoiding ultrasounds
- Researching birth centers and scheduling trips
- Interviewing obstetrical care providers
- Hiring a doula for labor and delivery
- Making preparations for a home birth
- Developing trust with obstetric health care clinicians
- Determining specific roles of supporters during labor and delivery
- Using TABS website for support
- Practicing hypnosis for childbirth
- Researching homeopathic remedies to prepare their body for childbirth

For three fourths of the women in our study, their subsequent birth was a healing one. Women felt empowered and said it brought reverence to the birthing process. As one woman shared, “I wasn’t made to feel like a piece of meat this time but instead like a woman experiencing one of nature’s most wonderful events” (Beck & Watson, 2010, p. 246). Women felt they had reclaimed their bodies and had a strong sense of control over birthing this time. All the attributes of a traumatic birth were absent this time around. Women felt respected. They were communicated with and felt cared for by the obstetrical staff. Sadly, not all women experienced a healing subsequent birth they desperately wanted. Some mothers purposely chose a home birth this time around to avoid all the medical intervention in the hospital. The women did give birth at home but due to postpartum hemorrhage needed to be transported to a hospital by ambulance.

## EMDR Treatment

Various treatments for PTSD in the general population have been studied for years. Researchers and clinicians are only starting to assess the effectiveness of these treatments for women who are suffering from PTSD due to traumatic childbirth. One study in my program of research focused on mothers’ experiences with EMDR treatment for their posttraumatic stress symptoms due to birth trauma (Beck et al., 2013). Ten women participated in this phenomenological study. All these mothers shared that this treatment was difficult but well worth the effort. As one woman shared, “I did EMDR for about 6 months and it helped me tremendously. It’s not that my memories or feelings about them are gone. But they are less excruciating and they don’t haunt me on a daily basis” (p. 185). Women who had experienced both cognitive therapy and EMDR treatment recalled that EMDR’s effectiveness in decreasing their posttraumatic stress symptoms was much faster than cognitive therapy.

## Does Traumatic Childbirth Affect More Than Just the Mother?

Evidence has accrued by researchers documenting the negative effects postpartum depression has on mother–infant interactions, infant’s cognitive and emotional development, and on women’s partners. The assumption I made was that posttraumatic stress in mothers who experienced birth trauma may also have a negative impact on their interactions with their infant and with their partners just as postpartum depression does.

## Partners

Fear, terror, and helplessness were emotions often recalled by partners who were present during the traumatic birth (Beck et al., 2013). One father painfully summed the experience as “I am on an island watching my wife drown and I don’t know how to swim” (p. 212). The relationships between women who had been traumatized at birth and their partners were negatively affected. Some women directed their anger at their significant others who had been present at the birth and did not advocate for them. Marital relationships were strained to the limit at times (Beck, 2004b). For instance, one woman revealed that

after 6 months, my husband and I still hadn’t had sex since before the birth. When we began to try, I had flashbacks to the birth. At the moment of penetration, I would have a flashback to the instant when my body was pulled down the operating table during one of the failed forceps attempts. (Beck, 2004b, p. 219)

## Mother–Infant Interactions

In my sustained research program on traumatic birth, four of the studies, three descriptive phenomenology and one case study, uncovered difficulties in mother–infant bonding and interaction. The impact of birth trauma on mother–infant interaction was not the primary focus of these studies. However, repeatedly insightful, troubling glimpses of the potentially detrimental impact of traumatic birth on infants’ cognitive and emotional development were revealed. In theoretical coalescence, this is one of the valuable advantages of being the researcher who has conducted all of the studies and is privy to all the data. Disturbing detachment from these infants came out loud and clear as some women avoided reminders of their birth trauma. Posttraumatic stress reared its ugly head here. For example, one mother stated that she felt as though her soul left her body leaving her an empty shell: “Mechanically, I’d go through the motions of being a good mother. Inside I felt nothing” (Beck, 2004b, p. 220).

The walls that traumatic birth erected separating mother and infant were not always temporary.

My child turned 3 years old a few weeks ago. I suppose the pain was not so acute this time. I actually made him a birthday cake and was grateful that I could go to work and not think about the significance of the day. The pain was less, but it was replaced by a numbness that still worries me. I hope that as time passes I can forge some kind of real closeness with this child. I am still unable to tell him I love him, but I can now hold him and have times when I am proud of him. I have come a long, long way. (Beck, 2004b, p. 222)

The intrusive flashbacks to the birth trauma led some women to experiencing interactions with their infants as a spectator. The “video tape” of the birth was continually on replay creating a bubble in which the mothers did not quite connect with anyone, not even their infants.

Detachment from their infants was reignited at anniversary time. The emotional bonding with their infants was missing. When describing her daughter’s first birthday, this mother admitted,

I wanted to die. I felt nothing for her and found it hard to celebrate the joy of this child that meant so little to me. I took excellent care of her but it was as if I was babysitting, the emotional bond just wasn’t there. (Beck, 2006b, p. 386)

Some women described looking in at their child’s birthday party from a window: Mothers felt “empty inside” and like a “total fake.” Traumatic childbirth also permeated mother–infant bonding during breastfeeding (Beck & Watson, 2008). Mothers described breastfeeding their infants as an “empty affair.” “Breastfeeding was just one of the many things I did while remaining totally detached from my baby” (p. 234). Posttraumatic stress left some mothers very disconnected from their babies as they breastfed.

In the descriptive phenomenological study of subsequent childbirth following a traumatic birth (Beck & Watson, 2010), the detrimental, chronic impact on mother–infant bonding was also revealed. Even if a woman had a healing subsequent birth she warned,

All the positive, empowering births in the world won’t ever change what happened with my first baby and me. Our relationship is forever built around his birth experience. The second birth was so wonderful I would go through it all again, but it can never change the past. (p. 247)

## Secondary Traumatic Stress

Secondary traumatic stress is the “natural consequent behaviors and emotions resulting from knowledge about a traumatizing event experienced by a significant other. It is the stress resulting from helping or wanting to help a traumatized or suffering person” (Figley, 1995, p. 10).

### Attributes/Characteristics

1. Intrusive flashbacks/nightmares
2. Numbing detachment
3. Increased arousal: anger, difficulty sleeping
4. Retreating from labor and delivery
5. Helplessness/powerlessness
6. Agonizing over what should have been

## Obstetrical Health Care Providers

When I would present the findings from my studies on traumatic childbirth and its resulting PTSD at conferences, at times labor and delivery nurses and CNMs would say to me, “You should study us. We are as traumatized as the mothers!” Based on these comments, I reviewed the literature and discovered the concept of secondary traumatic stress. I designed two mixed-methods studies to investigate the question: whether the impact of traumatic births extends to yet another group of persons: the health care providers who attended these births. The answer was a definite yes with both groups of nurses. Twenty-six percent of labor and delivery nurses screened positive for meeting all the diagnostic criteria of the *DSM-IV-TR* for PTSD due to traumatic childbirth (Beck & Gable, 2012). In the CNMs’ study, 36% screened positive for meeting all the diagnostic criteria for PTSD (Beck et al., 2015).

As seen in Table 3, labor and delivery nurses and CNMs also experienced four of the attributes of posttraumatic stress just as mothers did. Intensive flashbacks to the traumatic births were often described. For example, one labor and delivery nurse revealed,

Whenever I hear a patient screaming I will flashback to a patient who had an unmedicated (not even a local) cesarean section and the wailing of a mother when we were coding her baby in the delivery room. I feel like I will never get these sounds/images out of my head even though they occurred more than 10 years ago. (Beck & Gable, 2012, p. 757)

Difficulty sleeping due to nightmares was also a regular occurrence for CNMs as vividly illustrated by this quote:

The baby must have been dead for 5 days or so as the skin was peeling badly and blistered. I felt like I was pulling off skin and worried I would pull off the head. For weeks I could not get pictures of that dead baby out of my mind and had difficulty sleeping due to nightmares. (Beck et al., 2015, p. 21)

Anger was another distressing emotion obstetrical nurses experienced. Nurses at times were angry at obstetricians who were “abusive” to laboring women and were unnecessarily rough during vaginal exams and during the deliveries. At other times nurses directed their anger at themselves for not being a stronger advocate for their patients during childbirth. Both labor and delivery nurses and CNMs felt a sense of powerlessness and helplessness when they were not able to protect their patients from a traumatic birth. These obstetric clinicians would agonize over what should have been. Did they do everything that they should have done? What could they have done differently? Numbness was also experienced as nurses described being in shock immediately after a traumatic birth.

Avoidance of reminders of traumatic births occurred. For some obstetrical nurses, avoidance took the form of leaving labor and delivery and transferring to the postpartum unit. Others left the clinical area totally and went to graduate school with the goal of working in academia. CNMs also made similar changes in their careers to avoid traumatic birth. Some midwives left full scope practice and no longer did deliveries. Others went into academic or administration while others left midwifery altogether.

**Figure 2.**
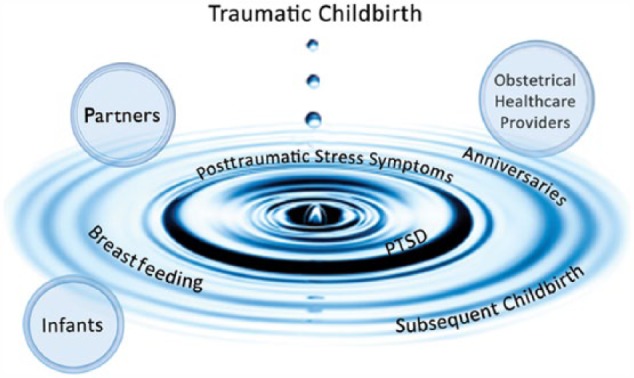
The ever-widening ripple effect. *Note.* PTSD = posttraumatic stress disorder. Note. Used with permission from iStock.

In conclusion, Figure 2 illustrates the theory of traumatic childbirth with its widening ripple effect not only on mothers but also on partners present during the birth trauma, obstetrical care providers who attended the births, and also on mother–infant interaction and bonding.

## Discussion

**Figure 3.**
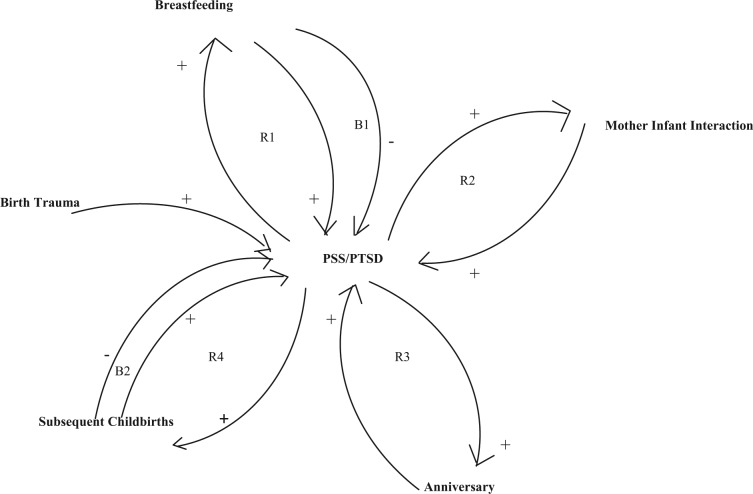
Amplifying causal looping in metaethnography of traumatic childbirth. *Source.* Reprinted with permission from Beck (2011, p. 307). *Note.* PSS = PTSD Symptom Scale; PTSD = posttraumatic stress disorder.

Using 6 of the 14 studies included in the development of this traumatic childbirth theory, Beck (2011) conducted a metaethnography of her qualitative research on birth trauma and its resulting PTSD. Key metaphors from each of the 6 studies were clustered into three overarching themes: stripped of protective layers, invisible wounds, and insidious repercussions. I used the concept of amplifying causal looping to further synthesize the findings from these 6 studies. In amplifying causal looping “as consequences become continually causes and causes continually consequences one sees either worsening or improving progressions or escalating severity” (Glaser, 2005, p. 9). Birth trauma resulted in six amplifying feedback loops: four of which were reinforcing and two of which were balancing (Figure 3). From the initial trigger of traumatic childbirth came these feedback loops which concentrated on lessening or worsening a mother’s posttraumatic stress symptoms. The metaethnography implications focused on leverage points where clinicians could intervene to try and break a feedback loop that produced undesired outcomes, namely, a worsening of posttraumatic stress symptoms.

This traumatic childbirth theory extends the findings of my metaethnography (Beck, 2011). The metaethnography concentrated on the worsening or improving posttraumatic stress symptoms that mothers experienced via the feedback loops. As that metaethnography was published, I have conducted additional studies in my research program such as, a qualitative study on fathers’ experiences being present during their partners’ traumatic births and mixed-methods studies on secondary traumatic stress in obstetrical health care providers. This traumatic childbirth theory reveals the chronic impact that just a short time in labor and delivery can have not only on the mothers themselves but also on their partners and infants. The midrange theory spotlights the ever-broadening rippling effect of traumatic childbirth, this time, on the partners and the clinicians who were present in the birthing room.

### Treatment

Debriefing, cognitive behavioral therapy (CBT), and EMDR therapy are three types of treatments available for PTSD. Research on these treatments for PTSD specific to traumatic childbirth, however, is limited.

#### Debriefing

Debriefing is referred to in the literature on a continuum. One end involves non–mental health care professionals, such as midwives, providing mothers an opportunity in an unstructured format to discuss their labor and delivery experience and to offer information and explanations about their birth. On the other end of the continuum is a structured psychological intervention given by trained mental health professions. This intervention is intended to serve as a primary prevention to help minimize symptoms of acute stress reactions. Mitchell and Dyregrov (1993) described the seven phases of debriefing: introduction, description of the events of the trauma, thoughts, emotions, symptoms, teaching, and last, a re-entry phase where the individual has an opportunity to ask any lingering questions and to develop a plan of action. Findings from limited research on the efficacy of different forms of debriefing for women with PTSD due to childbirth are not consistent. Conclusions cannot be drawn from these studies due to variations in methodological rigor and differences in questionnaires used to measure posttraumatic stress symptoms, in types of interventions, in timing when the debriefing was done, and in the duration of the debriefing.

#### CBT

In the general population, CBT is one of the most studied interventions for PTSD (Institute of Medicine, 2012). CBT focuses on problem-based interventions that consist of both a behavioral and a cognitive intervention. The behavioral intervention concentrates on minimizing maladaptive behaviors and increasing adaptive ones. This involves modifying antecedents and consequences. Next, behaviors are introduced that lead to new learning. The cognitive intervention focuses on changing maladaptive cognitions or beliefs. CBT in PTSD due to traumatic childbirth is the intervention that has been studied the least. Two case studies of mothers with postpartum PTSD who were successfully treated with CBT were described by Ayers, McKenzie-McHarg, and Eagle (2007).

#### EMDR

Shapiro (2001) developed EMDR treatment for PTSD. This treatment is based on the belief that traumatic events can get locked in the nervous system along with the original emotions and thoughts when the traumatic event happened. In EMDR treatment, while the person concentrates on the traumatic event, bilateral stimulation such as, eye movements back and forth, seems to assist in unlocking the nervous system, thus allowing the brain to process unconscious material on the trauma. During EMDR, the person learns to replace negative cognitions of self with positive cognitions of self when thinking of the traumatic event.

In the general population, EMDR has been effective in treating PTSD. Only two studies have been published that investigated this treatment with women with PTSD secondary to birth trauma (Sandstrom, Wiberg, Wikman, Willman, & Hogberg, 2008; Stramrood, van der Velde, Doornbos, Paarlberg, & Weijmar Schultz, 2012). These two pilot studies consisted of small samples of four mothers or less suffering from PTSD, though the researchers reported that these mothers experienced a decrease in their posttraumatic stress symptoms.

Lapp, Agbokou, Peretti, and Ferreri (2010) reviewed research on treatments of PTSD following traumatic childbirth and concluded that the effectiveness of debriefing was inconclusive but CBT and EMDR may decrease posttraumatic stress symptoms in mothers, although randomized control trials are needed.

### Implications for Clinical Practice

Three decades ago, when discussing nursing care in a Newborn Intensive Care Unit (NICU), Golub and Loizzo (1985) warned that “just as a stone thrown into water ruffles the surface in an outward direction, the feelings that arise in clinical situations generate emotional ripples of varied intensity” (p. 333). They went onto describe how the intense emotions that patients experience in clinical situations can “ripple out to affect everyone involved with their care” (p. 337). This midrange theory of traumatic childbirth confirms the widening ripple effect, this time with childbearing women.

Health care providers’ acts always have consequences on patients, either positive or negative. These consequences can spread out like ripples when a stone is dropped into a pond. Obstetrical clinicians need to ask themselves,

How far do your ripples go, and what are you willing to do to make sure those who are influenced by you are influenced in a positive way? It’s your call, it’s your life. But those ripples impact us all. The pond of humanity encompasses us all. Please be careful of your actions. (philosiblog.com)

Obviously, the best way to prevent PTSD secondary to traumatic childbirth is to prevent birth trauma in the first place whenever it is possible. During labor and delivery, women need to feel truly cared for, to be treated with respect, to be communicated with, and to have their dignity upheld. These protective layers may help prevent women from perceiving their childbirth as traumatic.

Obstetrical health care providers need to enact a proactive role in helping to prevent traumatic births. Knowing predictors of PTSD due to childbirth, such as high levels of medical intervention, is essential so that clinicians will be alert to these high risk women. As women are admitted to labor and delivery, health care professionals should take a detailed history regarding any fears women may have concerning giving birth. If a woman is multiparous, her admission history should address whether her previous births were considered traumatic. If so, this woman may be at high risk for being retraumatized during this current labor and delivery.

During the immediate postpartum period, clinicians need to be vigilant in observing new mothers for any signs and symptoms indicating they may have experienced a traumatic birth. Recognizing early trauma-related symptoms, such as a dazed look, withdrawal, or temporary amnesia, can be key to getting mothers necessary early interventions. Prior to discharge from the postpartum unit, health care providers need to explore with women if they perceived their birth was traumatic. Mothers’ unmet expectations concerning childbirth should be explored by clinicians as women’s perceptions of a traumatic experience may be based not only on the birth trauma itself but also on their unmet expectations regarding their anticipated birth process.

Reliable and valid instruments that screen for posttraumatic stress symptoms are available for clinicians’ use. The Post-Traumatic Stress Symptom Scale (Foa, Riggs, Dancu, & Rothbaum, 1993) is one such scale. If mothers do screen positive, referrals can be made for mental health care follow up. Mothers can also be given information about TABS which is a charitable trust located in New Zealand providing essential information and support to mothers regarding traumatic childbirth and its resulting PTSD (http://www.tabs.org.nz). Partners of women who have had a traumatic birth also need the attention of obstetrical care providers. Often partners who have been present during a traumatic birth are the forgotten ones.

While women are still on the postpartum unit, it is a perfect opportunity to observe mothers’ interactions with their infants that may indicate the women have experienced a traumatic birth. For example, when a mother is feeding her infant, does she appear distanced and detached? For some women with elevated posttraumatic stress symptoms, their infants can be reminders of their trauma leading to fragile mother–infant dyads that are in need of our help. Further complicating mother–infant bonding can be the numbness some mothers experience due to their birth trauma. Impaired mother–infant interactions may be one of the first indicators of a traumatic birth. Clinicians need to be cognizant that birth trauma can not only negatively affect mothers but can also have damaging impact on the beginning mother–infant relationships. Early intervention is critical.

Women who perceived that their birth was traumatic may need intensive one-on-one support as they initiate breastfeeding. It is essential that clinicians always ask the mother’s permission before touching her breasts to help with breastfeeding. Of course, providing support and encouragement to traumatized women to continue breastfeeding is important. Obstetrical care providers, however, also have a responsibility to let these struggling mothers know that it is their right to choose not to breastfeed without any guilt or judgment. If clinicians’ pressure traumatized mothers to continue breastfeeding, this can compound women’s feeling of inadequacy and shame and be detrimental to this already fragile mother–infant relationship.

If women go on to have another pregnancy after a previous traumatic birth, obstetrical care providers have a golden opportunity to help these traumatized women to reclaim their bodies and complete their journey to motherhood. The first step in achieving this, however, is to identify these women who have had a previous traumatic birth. During the first prenatal visit multiparas should be asked about their prior labor and deliveries. Giving traumatized women permission and encouragement to share their prior trauma is the beginning to helping them heal and to prepare for their upcoming childbirth. Unresolved trauma issues can be dealt with during this pregnancy. Clinicians can share with the mothers some of the strategies that women in Beck and Watson’s (2010) subsequent childbirth study described that helped them through the 9 long months of pregnancy waiting for the birthing process to begin. A subsequent birth can either help heal or retraumatize a mother. Clinicians have an essential role in determining which direction the mother’s subsequent birth will lead her.

Obstetrical clinicians failed to rescue women who perceived their birth was traumatic and we fail time and time again to rescue these mothers during their chronic struggles during yearly anniversaries and during subsequent childbirth. Often precious opportunities to finally rescue these women are lost. At infants’ well-baby checkups and yearly physical exams, health care professionals can ask mothers how they are doing and how they perceived their childbirth was. Pediatric clinicians are in an ideal position to identify women struggling with elevated posttraumatic stress symptoms and to make those critical referrals for mental health care.

### Suggestions for Future Research

There is such a paucity of research on traumatic childbirth that it can take multiple directions. Instruments developed to screen for posttraumatic stress symptoms in new mothers need to have their psychometrics compared. Intervention studies are needed to decrease the posttraumatic stress symptoms not only in mothers but also in their partners and clinicians who were present during the traumatic births. Randomized control trials testing the most effective treatments for PTSD due to childbirth are critical. Another intervention study can focus on improving mother–infant interaction during the time period mothers are struggling with the aftermath of their birth trauma. An example of one specific research question can be, “Does traumatic childbirth have similar disruptive relations in mother–infant dyads as postpartum depression does?” Another line of research can focus on the comorbidity of PTSD following childbirth with depression and other mood and anxiety disorders. Researchers also need to delve into the complex dynamics of traumatic birth on women’s breastfeeding experiences.

In conclusion, there is still much work that needs to be done on this traumatic childbirth theory. I will leave you with a quote from Highley and Mercer (1978) that reminds us all of the reverence that obstetrical care providers need to provide laboring women in hopes of preventing birth trauma and its ever-widening ripple effect:

Being able to assist a woman in one of the greatest tasks of her life—giving birth to and mothering a baby—is a privilege and challenge that touches every nurse who assists in her care. The challenge extends not only to the concrete physical help that the mother needs, but to the subtle consideration and attention which help her maintain her self-control and thus her self-respect. (p.41)
